# Immunomodulatory effects of the herbicide glyphosate following occupational exposure

**DOI:** 10.1007/s00204-025-04156-3

**Published:** 2025-09-08

**Authors:** Ambra Maddalon, Valentina Galbiati, Melissa Ferrian, Giuseppe Mastrangelo, Simone Meroni, Laura Dioni, Stefan Mandić-Rajčević, Claudio Colosio, Emanuela Corsini, Stefano Porru

**Affiliations:** 1 Laboratory of Toxicology, Department of Pharmacological and Biomolecular Sciences “Rodolfo Paoletti”, Università Degli Studi di Milano, Via Balzaretti 9, 20133 Milan, Italy; 2 Section of Occupational Medicine, Department of Diagnostics and Public Health, University of Verona, Verona, Italy; 3 Department of Cardio-Thoraco-Vascular Sciences and Public Health, University of Padua, Padua, Italy; 4 Department of Clinical Science and Community Health, Università Degli Studi di Milano, Milan, Italy; 5 Occupational Health Unit, International Centre for Rural Health, ASST Santi Paolo E Carlo, Department of Health Sciences, Università Degli Studi di Milano, Milan, Italy; 6 MISTRAL, Interuniversity Research Centre ‘Integrated Models of Study for Health Protection and Prevention in Living and Working Environments’, University of Brescia, Milano Bicocca and Verona, University of Verona, 37134 Verona, Italy

**Keywords:** Glyphosate, Occupational exposure, Immunomodulation, Cytokines, Lymphocytes, MiRNAs

## Abstract

Glyphosate, a widely used herbicide, has raised concerns regarding its impact on human health and the environment due to its widespread and excessive use. Adverse effects on the immune system have been reported. In this study, 26 vineyard workers in Veneto vineyards were examined before and after glyphosate applications to investigate possible immune parameter changes. Glyphosate exposure led to alterations in plasma cytokine levels, including marked increases in IL-4 and IL-5, a modest rise in IFN-γ, and a decrease in IL-8. No changes in plasma IL-12/23p40, IL17 and IL-33 were found. The IFN-γ/IL-4 ratio decreased, accompanied by changes in T cell subpopulations. Notably, a decrease in the T helper 1/T helper 2 cell ratio, attributed to reduced Th1 cells and increased Th2 cells, was observed, aligning with the elevated Th2 cytokines. A reduction in plasmatic extracellular vesicular miR-500a levels following glyphosate exposure was found, potentially contributing to the immunological findings. A slight decrease in plasma lipopolysaccharide levels in exposed workers excluded systemic inflammation from increased intestinal permeability but hinted at a possible association with glyphosate-induced microbiota dysbiosis. Since the shikimate pathway, targeted by glyphosate, is also present in Gram-negative bacteria. Overall, these findings suggest that glyphosate can affect the immune system, favoring Th2 responses. While more research is needed to establish causality with the association of glyphosate exposure with the development of allergic reactions in susceptible individuals, our results shed light on potential underlying mechanisms.

**Supplementary Information:**

The online version contains supplementary material available at https://doi.org/10.1007/s00204-025-04156-3.

## Introduction

Glyphosate is the active ingredient of one of the most widely used herbicide, mainly known by the brand name of Roundup^®^ (Benbrook 2016; Davoren and Schiestl 2018; Duke and Powles 2008). Glyphosate-based herbicides are commercialized in more than one hundred countries (Antier et al. 2020; Williams et al. 2000). It has been calculated that, in 2014, more than 113 million kg of glyphosate were agriculturally spread in the United States only (Benbrook 2016). The wide use of glyphosate was primarily due to its reputation of non-toxic for mammals, due to the absence of the shikimate pathway, which is the target of glyphosate (Höllander and Amrhein 1980). Despite this, evidence of toxicity in mammals is now present (Myers et al. 2016). In general, glyphosate exposure has been associated with cancer and neurological diseases, within others (Andreotti et al. 2018; Fortes et al. 2016; Rueda-Ruzafa et al. 2019; Schinasi and Leon 2014). Although there are discrepancies in the evidence and therefore also international agencies are not in accordance. Since agriculture is the main field of application of glyphosate, environment is one of the main sources of exposure. Few studies evaluated the effects of glyphosate occupational exposure in agricultural settings, like the Agricultural Health Study (Chang et al. 2023; Chatzi et al. 2007; De Roos et al. 2005; Slager et al. 2009), but none evaluated the effect of glyphosate-based herbicides alone. In this study, we focused on the immune system, since literature contains several possible associations between glyphosate and immune disorders (Peillex and Pelletier 2020). In particular, in vitro and in vivo studies highlighted altered cytokine production, lymphocytes response and subsets (Buchenauer et al. 2022; Kumar et al. 2024; Maddalon et al. 2022). Of particular interest, an in vivo study on mice revealed glyphosate action in promoting T helper 2-related cytokines (Kumar et al. 2024). We previously demonstrated that glyphosate can directly affect T cells, altering T cell differentiation and cytokine production. This includes a reduction in the Th1/Th2 ratio, primarily due to a decrease in Th1 cells, along with an increase in IL-4 and IL-17A production and a decrease in IFN-γ levels (Maddalon et al. 2022). Human epidemiological data further indicate that glyphosate could be associated with inflammatory diseases, like asthma and allergy (Chatzi et al. 2007; Hoppin et al. 2008 and 2017; Slager et al. 2009). Therefore, T helper cells and associated cytokines are the main focus of this research.

In view of this, the aim of the present study was to assess if glyphosate occupational exposure could affect immune parameters in vineyard workers. Blood samples were taken before and after glyphosate spray season, choosing workers not using other pesticides. Several cytokines and lymphocytes populations were evaluated together with lipopolysaccharide (LPS) plasma levels evaluation. In addition, the expression of different microRNAs (miRNAs) in plasmatic extracellular vesicles was also evaluated to get insights into the molecular mechanism of action.

## Materials and methods

### Study design

In this study, 26 male volunteers (25 vineyard worker and 1 fruit grower) working in 25 farms located in the Verona area were enrolled within the framework of a specific study (Porru et al. 2024). The average age in 2018 was of 48.2 year ± 12.4. The workers selected were not occupationally exposed to glyphosate or other pesticides over the six months before the study and those with chronic degenerative diseases were excluded. At the beginning of the study, following a clinical examination, two blood samples at two different times during the period between March and October 2017 were collected before the application of glyphosate. The second blood sample was collected 24 h after the last application of the season between March and July 2018. Blood samples were collected using BD Safety-Lok™ syringes (BD Biosciences, Becton, Dickinson and Company, New Jersey) and BD Vacutainer^®^ Blood Collection Tubes, using sodium citrate as an anticoagulant. Blood was frozen in cryotubes (Corning, New York) at −80 °C, following the addition at a ratio 1:1 of DMSO 20% in Sorensen phosphate buffered (0.133 M Na_2_HPO_4_ + 0.133 M KH_2_PO_4_, pH 7.2). To obtain plasma samples, blood was centrifuged at 1800×*g* at 4 °C for 15 min and supernatants centrifuged again at 3000×*g* at 4 °C for 15 min. A total of 52 blood and 52 plasma samples were available (26 pre-exposure and 26 post-exposure). All the samples were kept at − 80 °C.

Blood was used to assess lymphocytes percentage, whereas plasma was used to determine the level of cytokines and the presence of extracellular vesicles miRNAs was also evaluated as described below.

Samples were coded and blind tested, and only at the end of the data collection pre- and post-exposure samples were revealed.

### Cytokine evaluation

Plasma samples were evaluated for cytokine content (IFN-γ, IL-4, IL-5, IL-8, IL-12/23p40, IL-17A, IL-33). IFN-γ, IL-4, IL-8, and IL-12/23p40 content was evaluated through the use of commercially available ELISA kits from ImmunoTools (Friesoythe), whereas IL-5, IL-17A, and IL-33 ELISA kit were bought from Affymetrix (eBioscience, Santa Clara). All the procedures were conducted following the producer’s instructions. The detection limits were the following: 15,625 pg/mL for IFN-γ and IL-12/23p40, 7,813 pg/mL for IL-4, IL-5, and IL-33, 3,906 pg/mL for IL-8 and 1.56 pg/mL for IL-17A. All the data obtained after the interpolation of optical density with the standard curve, using the SpectraMax^®^ ABS (Molecular Devices, San Jose), are expressed in pg/mL of cytokine.

### LPS concentration evaluation

ELISA assay for LPS content in plasma samples was performed according to the manufacturer’s (MyBioSource, San Diego). The detection limit for LPS was 1.56 ng/mL.

### Lymphocytes analysis

Blood samples were analyzed for the percentage of lymphocyte subpopulations. In detail, CD3^+^CD4^+^ cells and CD3^+^CD8^+^ were evaluated using antibodies from Affymetrix eBioscience (APC-anti-human CD3, PE-anti-human CD4, and FITC-anti-human CD8). In brief, 200 μL of blood samples were transferred into flow cytometrical tubes (Greiner Bio-One, Kremsmünster) and 5 μL of each antibody diluted in 35 μL of staining buffer were added. Following a 30-min incubation at room temperature, avoiding direct light, 2 mL of 1-step Fix/Lyse Solution (eBioscience) were added and incubated for 15 min at room temperature. After that, the flow cytometrical tubed were centrifuged (500×*g*, 25 °C, 5 min) and the obtained pellet was diluted with 2 mL of staining buffer. 100 μL of cell suspensions were analyzed and the percentage of cells positive to CD3, CD4, and CD8 was evaluated using NovoCyte 3000 (Acea Biosciences, Agilent Technologies, Santa Clara). A pool of unstained samples composed of different workers’ blood was used to determine the negativity/positivity of markers.

Within CD4^+^ cells, corresponding to T helper (Th) cells, the percentage of Th1 (CD4^+^IFN-γ^+^), Th2 (CD4^+^IL-4^+^), and Th17 (CD4^+^IL-17^+^) was evaluated using the Human Th1/Th2/Th17 Phenotyping Kit (PharMingen, BD, New Jersey). For this experiment, whole blood was stimulated with phorbol 12-myristate 13-acetate (PMA—CAS# 16561-29-8) and ionomycin.) and ionomycin (CAS# 56092-82-1). In brief, 200 μL of blood samples were stimulated with PMA (50 ng/mL) and ionomycin (1 µg/mL), both purchased from Sigma-Aldrich (St. Louis), together with GolgiStop™ Protein Transport Inhibitor (PharMingen) for 5 h, following manufacturer’s instructions. After that, fixation, permeabilization, and staining procedure with PerCP-Cy5.5-anti-human CD4, PE-anti-human IL-17A, APC-anti-human IL-4, and FITC-anti-human IFN-γ were performed. 100 μL of cell suspensions were analyzed and the percentage of cells positive to IFN-γ, IL-4, and IL-17, within CD4^+^ cells was analyzed using NovoCyte 3000 (Acea Biosciences). A pool of unstained samples composed of different workers’ blood was used to determine negativity/positivity of markers.

### Analysis of miRNAs contained in extracellular vesicles

Extracellular vesicles were isolated from 52 plasma samples. In brief, 1 mL of plasma was transferred to a Microfuge^®^ Tube Polyallomer (Beckman Coulter, Brea). Plasma was ultracentrifuged using Optima™ MAX-XP Ultracentrifuge (Beckman Coulter) using TLA 100.3 rotor (Beckman Coulter) for 2 h at 100,000×*g* at 4 °C. Then the supernatant was discarded, the samples left at room temperature for 2 min, 700 µL of QIAzol^®^ Lysis Reagent (Qiagen) was added and the samples were conserved at − 80 °C. RNA extraction from extracellular vesicles was performed following the manufacturer’s instructions (miRNeasy^®^ Mini Kit, Qiagen, Hilden) and the RNA concentration was assessed using the NanoValue Plus (GE HealthCare Life Sciences, Boston) and samples were retro-transcribed using miScript II RT Kit (Qiagen). Before Real-Time PCR, the selected miRNAs were pre-amplified using the miScript PreAMP PCR kit (Qiagen). For PCR analysis miScript SYBR^®^ Green PCR Kit (Qiagen) was used. The quantification of the miRNAs was performed by the 2^−ΔCt^ method. The fold-changes of 7 miRNAs (hsa_miR-10b-5p, hsa_miR-27a-5p, hsa_miR-100-5p, hsa_miR-136-5p, hsa-miR-424-5p, hsa_miR-500a-5p, hsa_let-7f-5p) were analyzed, and miRNA hsa-RNU6-2 was used for normalization. The selection of the miRNAs to be assessed was driven by an initial screening on 3 pre-exposure and 3 post-exposure plasma samples performed with the QuantStudio 12 K Flex OpenArray^®^ system (Thermo Fisher Scientific, Waltham). 754 human miRNAs were amplified in each sample together with 4 internal controls (ath-miR159a, RNU48, RNU44 and U6 rRNA). MiRNAs with a Crt value > 28 or AmpScore < 1.1 or missing were considered unamplified. 12 miRNAs resulted to be statistically significantly different between pre- and post-exposure. We selected 6 of them (hsa_miR-10b-5p, hsa_miR-27a-5p, hsa_miR-100-5p, hsa_miR-136-5p, hsa-miR-424-5p, hsa_miR-500a-5p, hsa_let-7f-5p), based on their involvement in the immune system.

### Data analysis

Data are expressed as concentration of cytokine (pg/mL) or LPS (ng/mL), percentage of lymphocytes belonging to different subpopulations and miRNAs regulation as 2^−ΔCt^ values. Before conducting a statistical analysis, the parametric distribution was assessed. Depending on the data distribution, either a paired t-test or a Wilcoxon test was used, as indicated in the figure legends. The statistical analyses were performed using GraphPad Prism 10.0.0 version. Differences were considered significant at *p* ≤ 0.05.

### Correlation analysis

Based on exposure and questionnaire data (Porru et al. 2024), a correlation analyses with the immune endpoints analyzed in workers blood and plasma samples was performed. The urine levels of glyphosate were available for 17 vineyard workers and the questionnaire data for 24 workers out of 26. 2 participants were not able to deliver urine samples post-exposure, therefore, the data regarding urine glyphosate content and questionnaire were not available. Furthermore, due to technical issues in the lab that analyzed the urine levels, 7 urine samples could not be analyzed. Being the majority of the data non-parametric, the correlation analysis was done through the non-parametric Spearman correlation, using GraphPad Prism 10.0.0 version.

## Results

### Effects of glyphosate on plasma cytokines

**Fig. 1:**
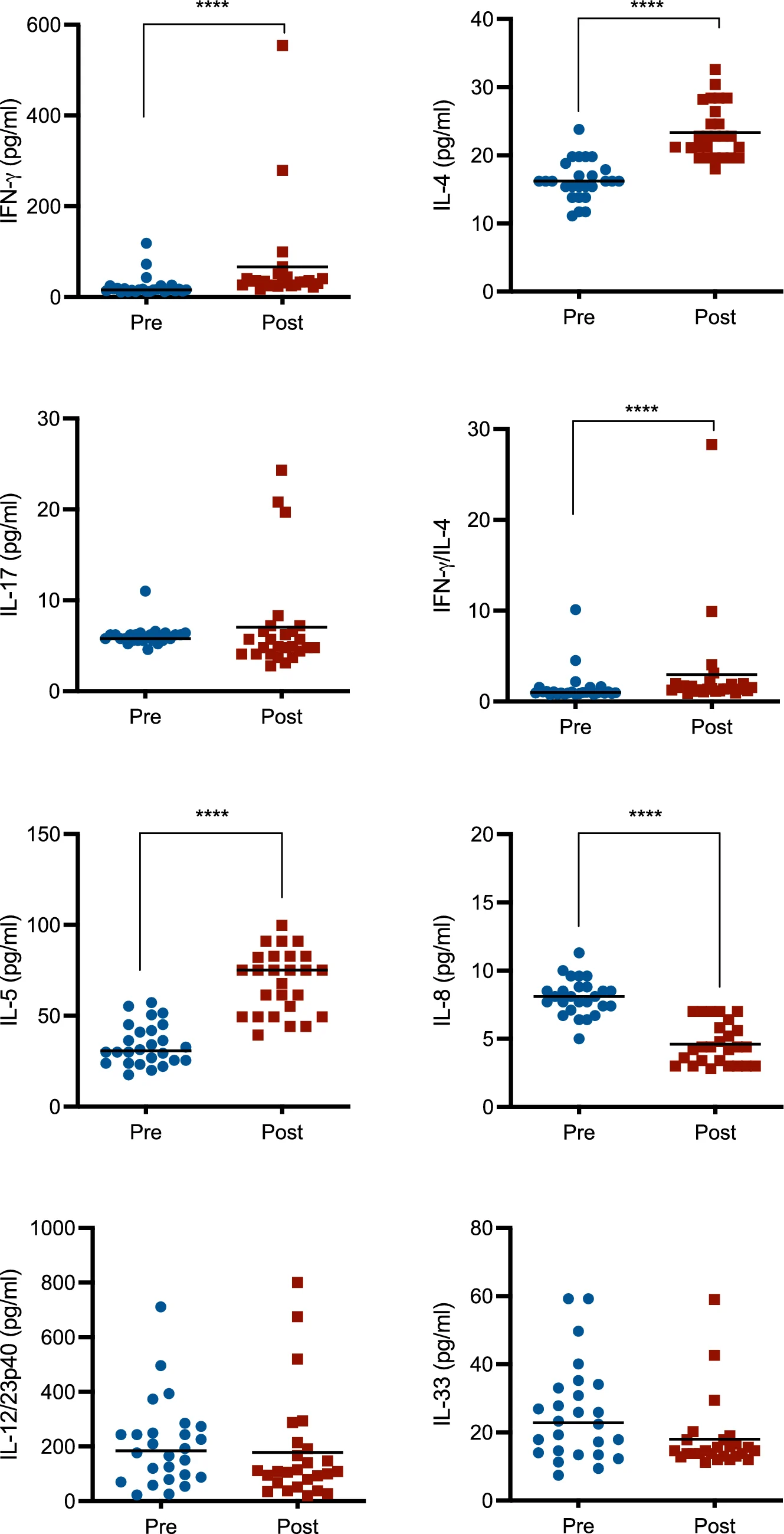
Effect of glyphosate occupational exposure on plasma cytokine levels. Pre- and post-exposure plasma samples were evaluated for cytokine content, namely IFN-γ, IL-4, IL-17, IL-5, IL-8, IL-12/23p40, and IL-33. Furthermore, the ratio IFN-γ/IL-4 was also calculated. Results are expressed as concentration (pg/mL) or ratio. Each blue dot indicates one pre-exposure sample, whereas each red square one post-exposure sample. The black line represents the median. Statistical analysis was performed with Wilcoxon test, with ****p ≤ 0.0001 Pre versus Post (colour figure online)

Glyphosate effects on the human immune system were first evaluated through the assessment of plasma cytokine levels (Fig. 1), to observe whether occupational exposure to the herbicide induced a dysregulation of immune mediators. In particular, we evaluated typical cytokines of T helper cells, namely IFN-γ, IL-4, and IL-17, and other cytokines, namely IL-5, IL-8, IL-12, and IL-33. Several differences were observed. Glyphosate was able to increase IFN-γ, IL-4, and IL-5 levels, whereas it decreased IL-8 plasma concentrations. Instead, no statistically significant changes in IL-17, IL-12, and IL-33 levels were detected upon glyphosate exposure. IFN-γ is a typical cytokine produced by T helper 1 cells and natural killer lymphocytes in response to different stimuli, like pathogens (Ivashkiv 2018). Furthermore, it stimulates B cells class switching and macrophage activation (Ivashkiv 2018; Su et al. 2015). IL-4 and IL-5 are typical cytokines produced by T helper 2 cells and mast cells and they also stimulate B cells proliferation (Eagar and Miller 2019). In particular, IL-4 increased production is linked to allergies (Gour and Wills-Karp 2015). IL-17 is the key cytokine released by T helper 17 cells, but also other cells have the ability to release it, and its main action is the defense against bacteria and fungi (Amatya et al. 2017). On the other side, IL-8 is a pro-inflammatory cytokine, mainly released by macrophages and several target cells can respond to this chemokine, like neutrophils, mast cells, lymphocytes, and keratinocytes (Brennan and Zheng 2007). IL-12 main action is to contribute to the development of T helper 1 cells, whereas IL-33 is a promoter of T helper 2 cells (Akdis et al. 2004).

### Impact of glyphosate on plasma LPS levels

**Fig. 2:**
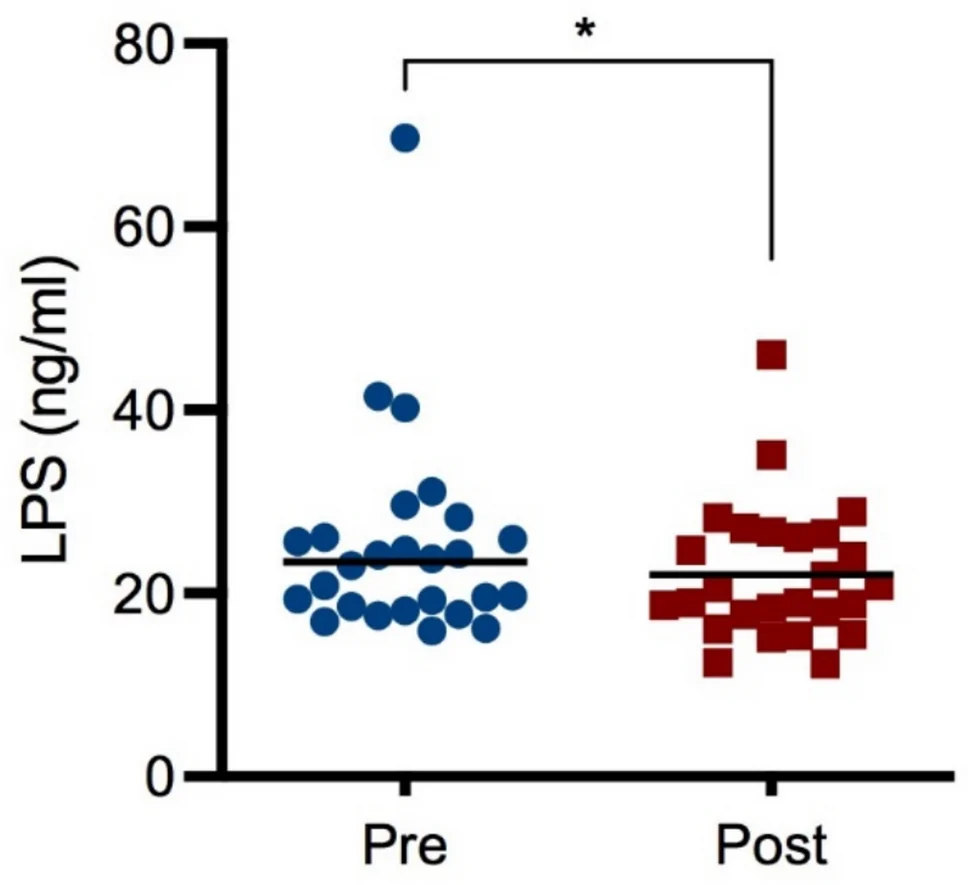
Effect of glyphosate occupational exposure on plasma LPS. Pre- and post-exposure plasma samples were evaluated for LPS concentration. Results are expressed as concentration (pg/mL). Each blue dot indicates one pre-exposure sample, whereas each red square one post-exposure sample. The black line represents the median. Statistical analysis was performed with Wilcoxon test, with *p ≤ 0.05 Pre versus Post (colour figure online)

As several intestinal microorganisms express the enzyme EPSPS, an attractive hypothesis could be the one that connects microbiota dysbiosis with possible changes in immune parameters. Gram-negative bacteria discharge LPS/endotoxin into the bloodstream through their growth, cell lysis following antibiotic treatment, or translocation from the gut (Mohr et al. 2022). LPS can be a potent stimulant of host immunity, but this response depends on the microbial species’ origin (Kumar et al. 2024). The plasma levels of LPS were measured by a commercial available ELISA (Fig. 2). A slight reduction of LPS levels in vineyard workers post-exposure samples was observed, which could explain the lower level of plasma IL-8 in post-exposure samples, and may be indicative of dysbiosis. The reduction of plasma LPS could be due to the ability of glyphosate to inhibit G- bacteria growth, as shown for most strains of *K. pneumoniae* and *E. coli* and the *E. cloacae* (Zerrouki et al. 2024). LPS changes can arise from various mechanisms. Indeed, glyphosate may affect gut barrier integrity, independently of significant changes in microbial composition (i.e., affecting tight junctions, increasing intestinal permeability).

### Effect of glyphosate exposure on T lymphocytes

**Fig. 3:**
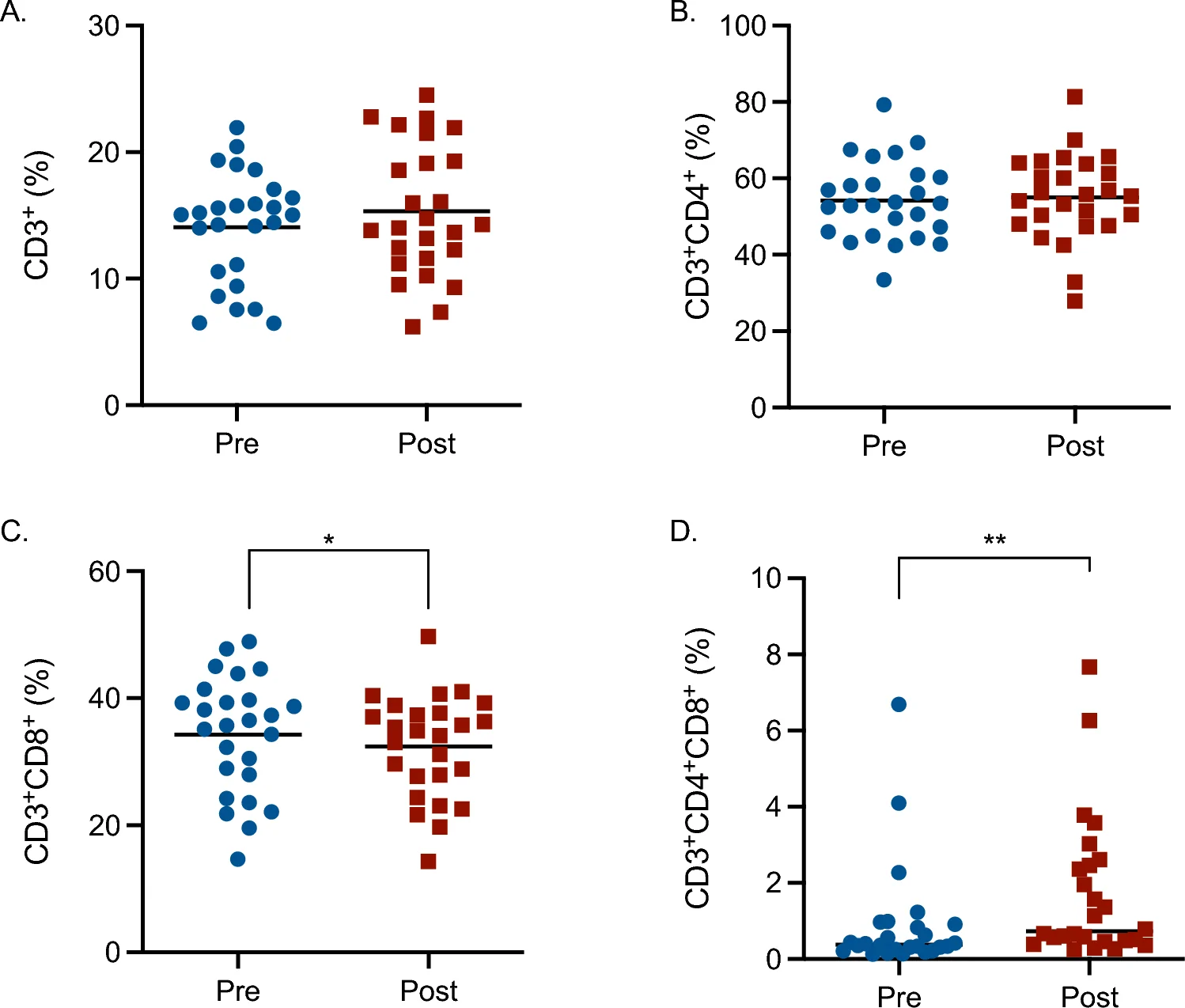
Effect of glyphosate occupational exposure on T lymphocytes expressed as percentage of CD3^+^ (**A**), CD3^+^CD4^+^ (**B**), CD3^+^CD8^+^ (**C**), and CD3^+^CD4^+^CD8^+^ cells (**D**). Pre- and post-exposure blood samples were evaluated for T lymphocytes. Each blue dot indicates one pre-exposure sample, whereas each red square one post-exposure sample. The black line represents the mean (with the exception of D, where it represents the median). Statistical analysis was performed with a paired *t*-test, with *p ≤ 0.05 Pre versus Post or Wilcoxon test for Panel **D**, with **p ≤ 0.01 Pre versus Post. The gating strategy is reported in Supplementary Fig. 1 (colour figure online)

Blood samples pre- and post-exposure were evaluated for the assessment of glyphosate effects on T lymphocytes (Fig. 3). Total T cells and the main classes (T helper cells—CD4^+^—and cytotoxic T cells—CD8^+^) were investigated. The percentage of T cells and of T helper cells (Fig. 3A, B) was not affected by glyphosate exposure, whereas cytotoxic T cells (Fig. 3C) resulted slightly reduced following glyphosate exposure, contrarily to double positive CD4^+^CD8^+^ cells (Fig. 3D), which resulted to be increased. A reduction of cytotoxic T cells is a feature of several autoimmune diseases and it is also indicative of a reduced immune response against pathogens (Pandher et al. 2021). Regarding double positive T cells (CD4^+^CD8^+^), there is proof identifying these cells in inflammatory and autoimmune disorders (Parel and Chizzolini 2004), nevertheless, more investigations are needed.

Furthermore, the total amount (cc/μL) of total T cells, T helper and cytotoxic T cells (Supplementary Table 1) are reduced after glyphosate exposure. Instead, the increasing trend in the percentage of double-positive CD4^+^CD8^+^ is reflected by the cc/μL values (Supplementary Table 1).

### Effects of glyphosate exposure on T helper cell subpopulations

**Fig. 4:**
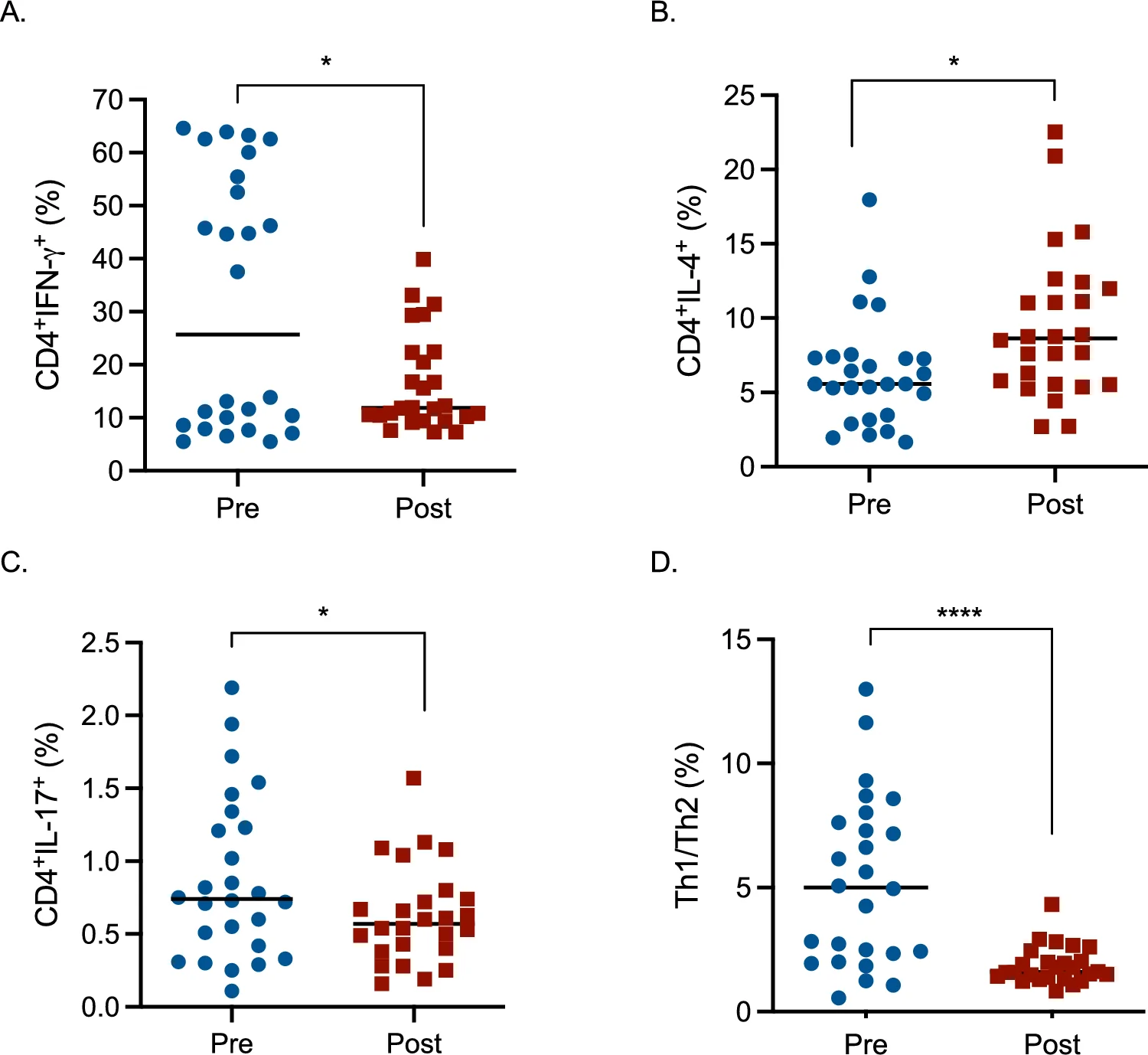
Effect of glyphosate occupational exposure on T helper cells differentiation expressed as the percentage of CD4^+^IFN-γ^+^ (**A**), CD4^+^IL-4^+^ (**B**), CD4^+^IL-17^+^ (**C**), and the ratio between Th1 (CD4^+^IFN-γ^+^) and Th2 (CD4^+^IL-4^+^) (**D**). Pre- and post-exposure blood samples were evaluated for T helper cells subpopulations. Each blue dot indicates one pre-exposure sample, whereas each red square one post-exposure sample. The black line represents the median. Statistical analysis was performed with the Wilcoxon test, with *p ≤ 0.05, ****p ≤ 0.0001 Pre versus Post. The gating strategy, together with representative histograms, is reported in Supplementary Fig. 2 (colour figure online)

Based on the dysregulation of several cytokines mainly produced by T helper cells (Fig. 1) and based on literature evidence suggesting the ability of glyphosate to impair T helper subsets (Kumar et al. 2014), the effect of occupational exposure on Th cells differentiation (Th1, Th2 and Th17 cells) was investigated (Fig. 4). A reduction of T helper 1 and 17 cells (Fig. 4A, C) and an increase of T helper 2 cells (Fig. 4B) were observed following exposure to glyphosate. The Th1/Th2 ratio was also reduced, indicating an unbalance toward Th2 responses. T helper 1 cells are the main actor against intracellular pathogens, and they can activate macrophages and neutrophils (Romagnani 1991). A decrease in this class of cells can, in turn, lead to a decreased ability to fight against infections. Also T helper 17 cells, which are involved in autoimmunity and tissue inflammation (Park et al. 2005), are reduced following glyphosate exposure. Contrarily, T helper 2 cells resulted increased. They are often thought as the counterpart of T helper 1 cells and they are involved in defense against parasites and they also have a role in allergic inflammation, like asthma (Zhu and Zhu 2020).

In addition, the amount of cells belonging to the T helper 1 population and the ratio between T helper 1 and T helper 2 (Supplementary Table 1) mirrored the reduction observed in the % data. Also T helper 2 increase is mirrored by cc/μL data. Contrarily, regarding the subpopulation of T helper 17, the % is decreased, whereas an increase in the concentration is noticed (Supplementary Table 1).

**Fig. 5:**
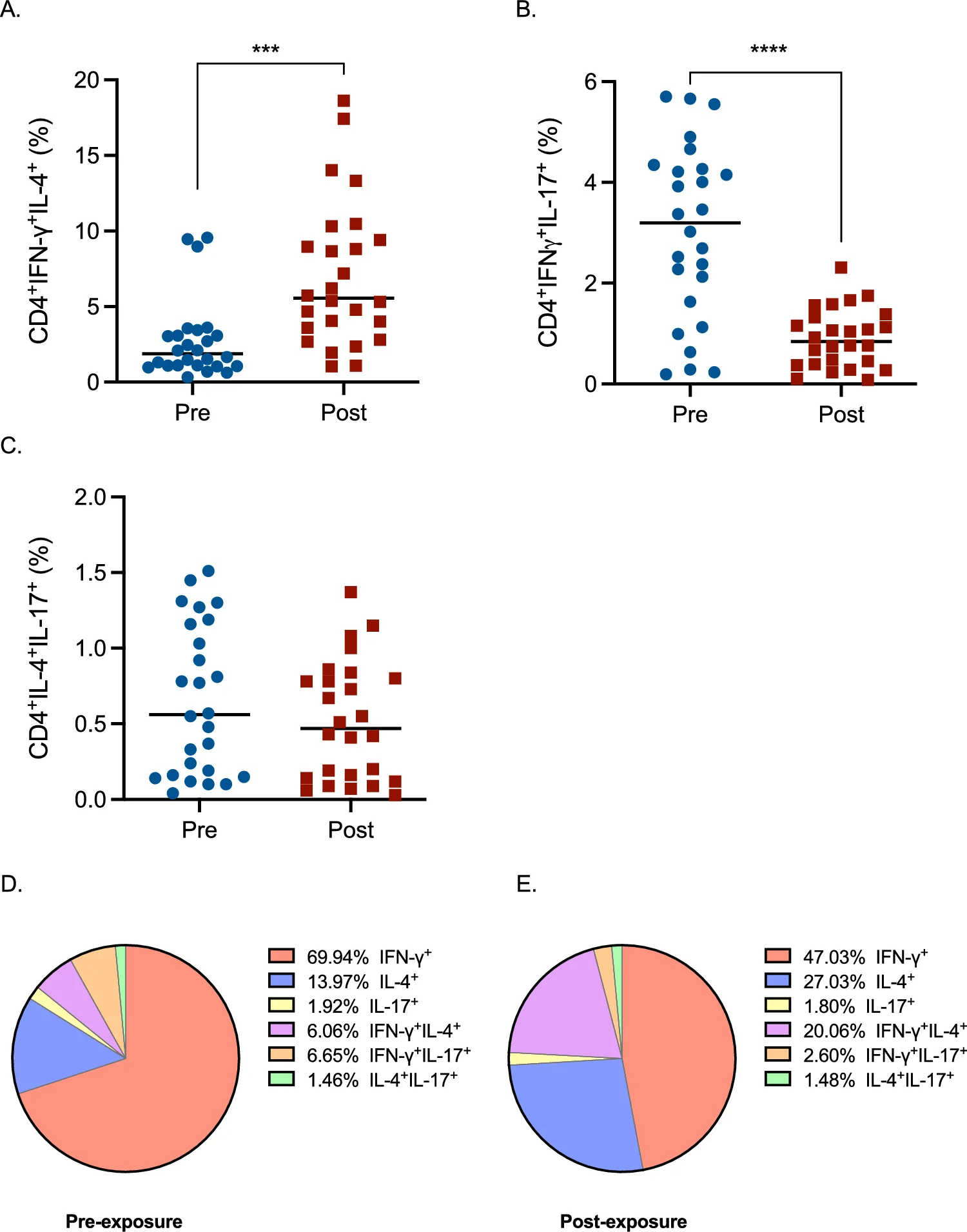
Effect of glyphosate occupational exposure on T helper cells subpopulations expressed as the percentage of CD4^+^IFN-γ^+^IL-4^+^ (**A**), CD4^+^IFN-γ^+^IL-17^+^ (**B**), CD4^+^IL-4^+^IL-17^+^ (**C**). Pre- and post-exposure blood samples were evaluated for T helper cells subpopulations. Each blue dot indicates one pre-exposure sample, whereas each red square one post-exposure sample. The black line represents the median. Statistical analysis was performed with Wilcoxon test, with ***p ≤ 0.001, ****p ≤ 0.0001 Pre versus Post. Panels **D** and **E** represent pie charts of the distribution of the subpopulations reported in Figs. 4 and 5 of all the pre- (**D**) and post-exposure (**E**) blood samples to visualize the effects of glyphosate on Th cells (colour figure online)

Double-positive subpopulations were also evaluated (Fig. 5). Analyzing double-positive populations can provide valuable insights into the complexity of T helper cell responses. These populations represent a mixed cytokine profile, which can reveal important aspects of cell plasticity and heterogeneity, and this will give a more complete picture of the overall activity of T helper cells. An increase in T helper cells double positive for IFN-γ and IL-4 was observed (Fig. 5A), while double positive cells for IFN-γ and IL-17 decreased (Fig. 5B). A slight trend of reduction was also observed for T helper cells double positive for IL-4 and IL-17 (Fig. 5C). The roles of double positive populations are not fully understood. Double positive (T helper 1–2 cells) are thought to be memory T helper cells with the normal ability to produce IL-4 that have also acquired the ability to produce IFN-γ (Krawczyk et al. 2007), and their modulation has the same trend as T helper 2 cells. The role of the other double-positive population has not been addressed up to now, but these cells might have intermediate properties.

Furthermore, the total amount of T helper cells double positive for IFN-γ and IL-4 (cc/μL) (Supplementary Table 1) reflected the increasing trend of the % values.

To sum up the effects of glyphosate on T helper cells subpopulations, a general reduction of IFN-γ^+^ and IFN-γ^+^IL-17^+^ cells together with an increase of IL-4^+^ and IFN-γ^+^IL-4^+^ cells can be observed in post-exposure samples (Fig. 5D, E).

### Effects of glyphosate on plasma extracellular vesicular miRNAs

**Fig. 6:**
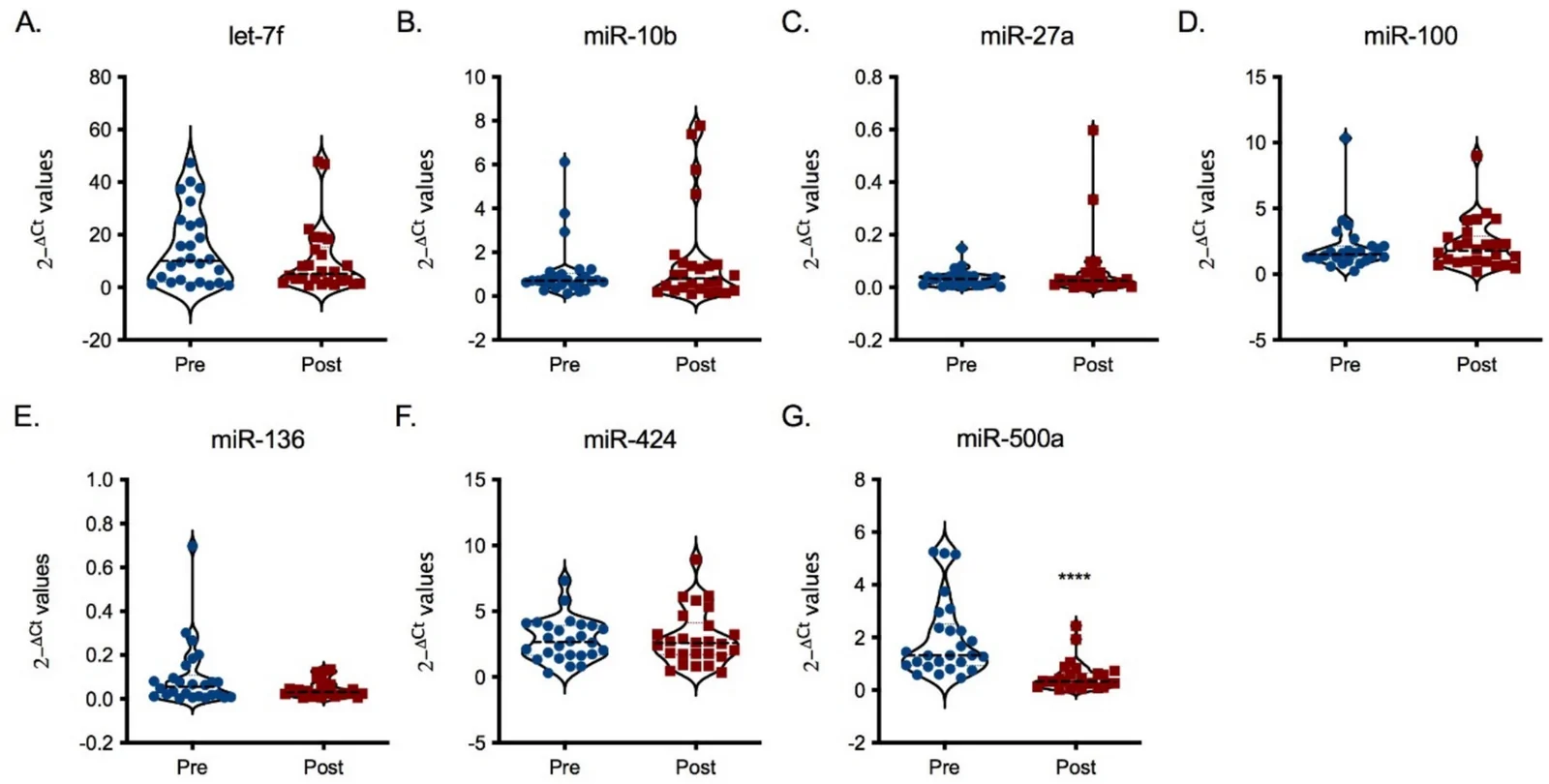
Effect of glyphosate occupational exposure on plasma extracellular vesicular miRNAs. Extracellular vesicles were extracted from plasma samples and miRNAs content evaluated by RT-PCR. miRNAs analyzed were let-7f (**A**), miR-10b (**B**), miR-27a (**C**), miR-100 (**D**), miR-136 (**E**), miR-424 (**F**), and miR-500a (**G**). Data are expressed as 2^−ΔCt^ values. Each blue dot in the violin plots indicates one pre-exposure sample, whereas each red square one post-exposure sample. The black line represents the median. Statistical analysis was performed with Wilcoxon test, with ****p ≤ 0.0001 Pre versus Post (colour figure online)

Finally, to have a possible understanding of the mechanism of action of glyphosate, miRNAs expression in extracellular vesicles was evaluated. Initially, a panel of 754 human miRNAs was evaluated in plasma samples obtained from 3 pre- and 3 post-exposure individuals, randomly chosen without pairing. 12 miRNAs resulted to be statistically significantly de-regulated following glyphosate exposure (Supplementary Table 2). From these, 7 miRNAs involved in the immune response were selected, and their expressions are shown in Fig. 6. Several trends in different expressions in miRNAs following glyphosate exposure could be observed, which showed statistical significance for the down-regulation of miR-500a (Fig. 6G). Recent data highlighted a role of miR-500a in the tuning of inflammatory response, and its downregulation is linked to elevated expression of several genes, such as NR3C1, KPNA1, EIF5, and CSF3 (Guo et al. 2023). Interestingly, some of these genes are involved in the immune response; CSF3 encodes for a member of the IL-6 superfamily of cytokines and NR3C is involved in inflammation repression.

### Correlation analysis between glyphosate application and immune endpoints

**Fig. 7:**
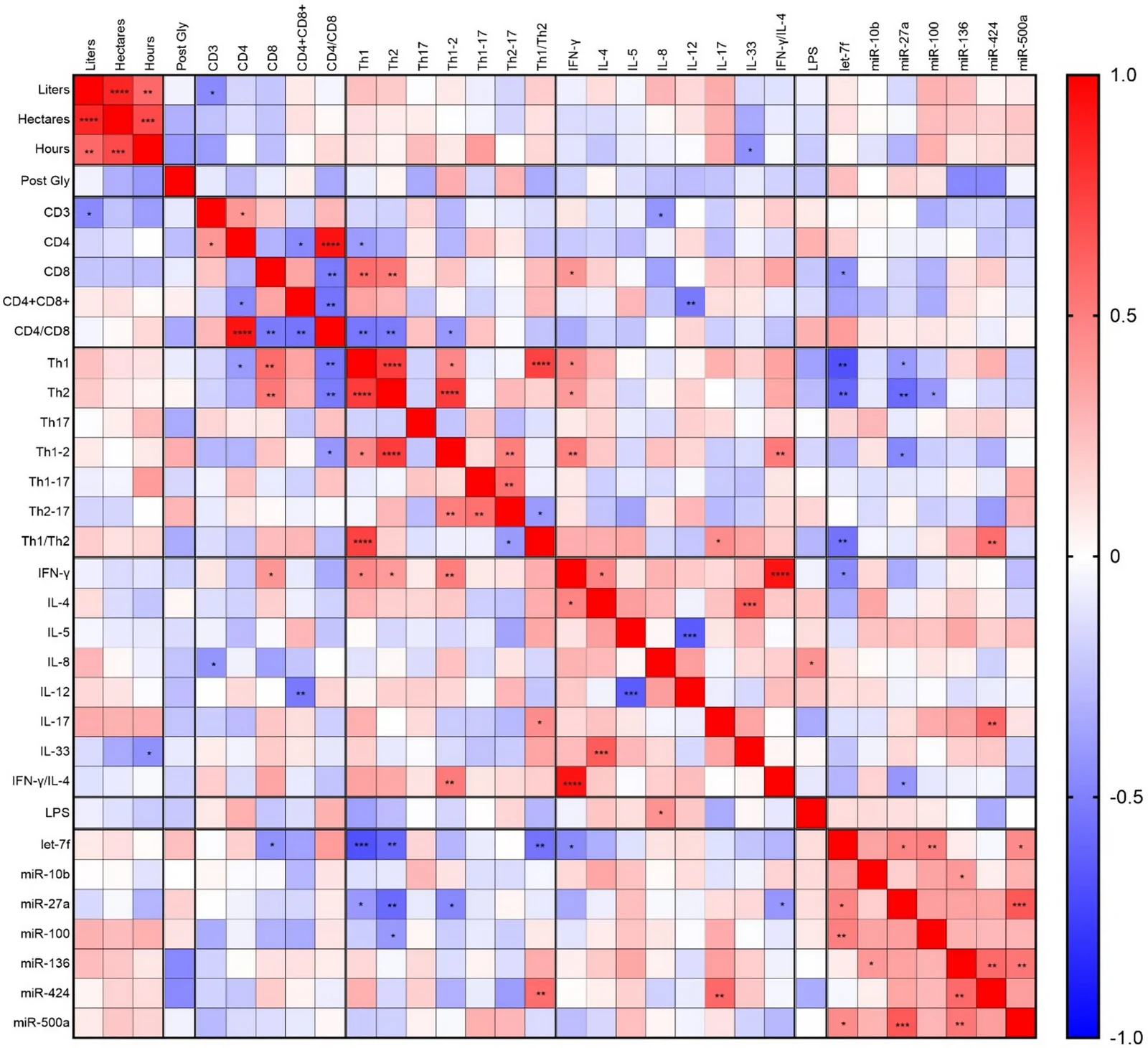
Correlation matrix between glyphosate exposure data and immune endpoints. Data obtained from vineyard workers’ questionnaires (liters of herbicide used—liters—, hectares treated with the herbicide—hectares—, and exposure time—hours), from urine analysis (evaluation of urine glyphosate levels post exposure—post Gly), and from the immune endpoints previously shown (ratio between post- and pre-exposure) were analyzed with non-parametric Spearman correlation. The heatmap shows Spearman *R* values (from blue − 1, meaning negative correlation, to red + 1, meaning positive correlation). All the *R* values and the *p* values are shown in Supplementary Excel File 1. *p ≤ 0.05; **p ≤ 0.01; ***p ≤ 0.001; ****p ≤ 0.0001 (colour figure online)

Based from exposure data published in Porru et al. (2024), a correlation analysis with the immune endpoints was performed (Fig. 7). A clear positive correlation between liters of glyphosate applied, hectares treated and hours of exposure to glyphosate can be observed as far as immune endpoints are concerned, which is however not reflected in the urinary levels of glyphosate, probably because of the use of personal protective equipments. Post-exposure glyphosate urine levels did not statistically significantly correlate with any of the immune endpoints. However, an overall consistency in the trend can be observed, with an inverse correlation with suppressed parameters and a positive association with parameters upregulated, with the exception of plasma IFN-γ and IFN-γ/IL-4 ratio. Some correlations among the immune endpoints can be observed, including cytokines, lymphocyte subpopulations and microRNAs. let-7f and miR-27a showed statistically significant negative correlations with several lymphocytes subpopulations and cytokines, mainly T helper 1, T helper 2, and IFN-γ/IL-4 ratio. Also miR-100 negatively correlated with T helper 2 percentages, whereas miR-424 positively correlated with T helper 1/T helper 2 ratio and with IL-17 levels. Although glyphosate did not alter the expression of these miRNAs in a statistically significant way, they could be involved in the underlying mechanisms of glyphosate-induced immune dysregulation.

## Discussion

The purpose of this work was to investigate potential immunomodulatory effects of glyphosate in occupationally exposed vineyard workers. Plasma and blood samples obtained pre- and after- glyphosate use were evaluated for cytokine levels, lymphocyte subpopulations, and extracellular vesicles miRNAs. Several parameters resulted altered after glyphosate exposure. Overall, results point out a skew towards a Th2 phenotype, with decreased Th1 and Th17 cells.

Exposure to glyphosate led to alterations in plasma cytokine levels, including marked increases in IL-4 and IL-5, a modest rise in IFN-γ, a decrease in IL-8, and no changes in plasma IL-12/23p40, IL17 and IL-33, suggesting selective effects and not generalized immune stimulation. Among the cytokines affected, the increase in plasma IL-4, and the reduced Th1/Th2 ratio following glyphosate exposure are of interest, as it is consistent with previous results we obtained in vitro on human peripheral blood mononuclear cells exposed to low concentrations of glyphosate (Maddalon et al. 2022). In vitro treatment also resulted in decreased IFN-γ and increased IL-17 production, which was not observed in vivo in the current study, where a slight increase in IFN-γ was observed and a trend toward increased IL-17 following exposure that did not reach statistical significance. The in vitro effect indicates that glyphosate can directly modulate T cell activation. In addition, the immunomodulatory effects of glyphosate can be prevented using ICI 182,780, indicating a role of estrogen receptor-α in the observed effects. Other studies in animals have investigated cytokine production, with consistent results observed. The increased IL-5 is consistent with results obtained in mice exposed to a pool of air samples obtained during the glyphosate spray season of three farms in Ohio (Kumar et al. 2014). Both IL-4 and IFN-γ resulted in increased female mice chronically exposed to glyphosate (Buchenauer et al. 2022).

Regarding lymphocytes, a decrease in the total amount of T cells, T helper and cytotoxic T cells was observed. In detail, lower T helper 1 and 17 cells and higher T helper 2 cells were found following glyphosate exposure. T helper 2 cells showed the same trend of IL-4 modulation, whereas T helper 1 cells decreased while IFN-γ slightly increased. This could be explained by the fact that the plasmatic cytokine levels do not derive only from lymphocytes but also from other cell types. The decrease in T helper 1 and in the ratio between T helper 1 and 2 mirrored our previously obtained results in vitro (Maddalon et al. 2022). The increased T helper 2 response could be linked to an increased risk of allergic and atopic responses (Berger 2000). Whereas a decreased T helper 1 and T helper 17 response might lead to a higher susceptibility to infections and autoimmune disturbances (Zambrano-Zaragoza et al. 2014). In vivo evidence of the action of glyphosate on these adverse immune-related health outcomes are present, namely increased lung inflammation, wheeze, and allergic rhinitis (Buchenauer et al. 2022; Chatzi et al. 2007; Hoppin et al. 2017; Islam et al. 2022; Lehman et al. 2023; Kumar et al. 2014; Pandher et al. 2021; Parks et al. 2016; Slager et al. 2009).

The skew towards the Th2 phenotype observed following glyphosate exposure is unlikely to be due to the airborne pollen season during which most of the second sampling occurred. It is important to mention that within the study group, four subjects reported being allergic to pollen. Excluding these individuals from the analysis did not alter the results or conclusions regarding the effects of glyphosate. Following glyphosate exposure, the lower Th1/Th2 ratio was observed in all subjects. The strongest argument against this explanation it is the demonstration of the direct effect of glyphosate in vitro on T cells that can reproduce many of the effects observed in the current study (Maddalon et al. 2021). Furthermore, in non-allergic individuals during pollen season modest or no changes in the immune status are reported. Unlike allergic individuals, non-allergic people often exhibit a regulatory or tolerogenic immune response, characterized by increased activity of regulatory T cells (Tregs) or anti-inflammatory cytokines like IL-10 (Akdis et al. 2004; Gökkaya et al. 2020; Shamji et al. 2019; Tamm et al. 2018). This emphasizes that the observed effects on immune parameters were specifically attributable to glyphosate, independent of pollen allergy status.

One hypothesis that might connect glyphosate to the observed adverse immune effects is the action mediated by microbiota dysbiosis (Maddalon et al. 2021). Indeed, some microorganisms, also present in gut microbiota, possess the shikimate pathway, which is the target of glyphosate in plants. There are several studies highlighting the ability of glyphosate to perturb gut microbiota (Aitbali et al. 2018; Lehman et al. 2023; Liu et al. 2022a; Mendler et al. 2020). The close relationship between microbiota and immune system has been demonstrated (Ranganathan and Sivasankar 2014) and therefore it could be possible that, through gut dysbiosis, glyphosate could induce adverse immune effects. The decrease in plasma LPS observed in farmers after exposure may indicate a possible dysbiosis. Since LPS can strongly stimulate host immunity—though this response varies depending on the microbial species of origin (Kumar et al. 2024)—further studies are needed to clarify the role of dysbiosis in glyphosate-induced immune alterations in humans. In addition to this hypothesis, a direct effect of glyphosate on human immune cells has been demonstrated, with findings consistent with current results.

Among the miRNAs present in microvesicles, miR-500a was down-regulated in post-exposure plasma samples. miRNAs are short non-coding RNAs with the role of gene expression post-transcriptional regulators (Ranganathan and Sivasankar 2014). Extracellular vesicles can be found in blood and other body fluids and they can be secreted by different cell types (Alberro et al. 2021). The miRNAs contained represent important information to be transported to the recipient cells/tissues, and could serve as important biomarkers (Schwarzenbach 2017). miR-500a is understudied, but the few literature data available evidenced it as a biomarker for systemic lupus erythematosus and hepatocellular carcinoma (Dai et al. 2009; Yamamoto et al. 2009). Furthermore, it is also involved in oxidative stress related to breast cancer (Degli Esposto et al. 2017) and it shows the ability to activate the nuclear factor κB, inducing cell proliferation (Zhang et al. 2015). Based on different databases able to predict mRNA targets, miR-500a-5p could be able to interact with several mRNAs involved in the immune responses, such as AHR, BCL10, CCR4, IKZF2, IL10RA, NFKB2, and STAT1. Several epigenetic changes induced by glyphosate have already been revealed (Bukowska et al. 2022; Liu et al. 2022b; Mesnage et al. 2022), but no studies evaluated them in human following occupational exposure. Interestingly, a recent study we performed to investigate the in vitro effects of glyphosate revealed an up-regulation of miR-500a both in exosomes as well as intracellular (Maddalon et al. 2022). The use of the inhibitor of hsa-miR-500a-5p prevented glyphosate-induced decrease in IFN-γ. Also the expression of miR-500a could be prevented by ICI 182,780. The different trend of modulation should have different explanations, such as time of exposure, or more probably miR-500a found in microvesicles of vineyard workers’ plasma samples derives from other cells/tissues that in an in vitro study are not present. The biological implications of the dysregulation of this miRNA must be further investigated.

The correlation analysis performed showed an overall consistency with the immunological parameters, even if post-exposure glyphosate urine levels do not statistically significantly correlate with any of the immune endpoints. Trends show an inverse correlation with suppressed parameters and a positive association with parameters upregulated. Furthermore, several miRNAs, in particular let-7f and miR-27a, resulted highly negatively correlated with other immune endpoints, highlighting the possible importance of these miRNAs in glyphosate immunomodulation, which requires further investigation. One of the main reasons for the low amount of correlations with post-exposure glyphosate urine levels could be the limited number of samples analyzed.

To conclude, occupational exposure to glyphosate was able to affect several immune parameters, with a skewed Th2 phenotype that might in turn lead to putative adverse health effects. This study included a limited number of workers, therefore, it would be relevant to confirm the data obtained in this study on a broader population. While perspective studies will be necessary to link acute glyphosate exposure with immune-mediated diseases. In light of the immunomodulatory properties exerted by glyphosate, several mechanistic studies more focused on the immune system should be performed.

## Supplementary Information

Below is the link to the electronic supplementary material.

Supplementary file1 (DOCX 235 KB)

Supplementary file2 (XLSX 32 KB)

## Data Availability

The data that support the findings of this study are available upon request to the authors.
