# Supplementary material for: Immunomodulatory effects of the herbicide glyphosate following occupational exposure
Source: Arch Toxicol. 2025 Sep 8;99(12):4973–86. doi: 10.1007/s00204-025-04156-3 (PMC12534365; doi:10.1007/s00204-025-04156-3)
Supplement: Supplementary file 1 — Supplementary file1 (DOCX 235 KB) [file 204_2025_4156_MOESM1_ESM.docx]

**Supplementary Materials**

Supplementary Figure 1. Gating strategy for the detection of CD3^+^ subpopulations (CD4^+^, CD8^+^, and CD4^+^CD8^+^ cells). The gate Lymphocytes is created from the FSC/SSC dot plot, and from this gate CD3^+^ cells are detected. From CD3^+^ cells, CD4^+^, CD8^+^, and CD4^+^CD8^+^ cells are detected.


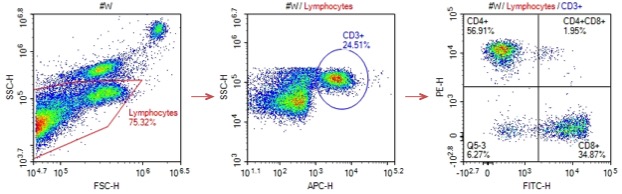


Supplementary Figure 2. Gating strategy for the detection of CD4^+^ subpopulations (IFN-γ^+^, IL-4^+^, and IL-17^+^ cells, together with double positive cells for the cytokines). The gate CD4^+^ is created from the FSC/SSC dot plot, and from this gate the different subpopulations are detected. On the bottom the overlays of the histograms of the expression of IFN-γ, IL-4, and IL-17 of one representative blood sample pre-exposure (blue) and one post-exposure (red) are shown.


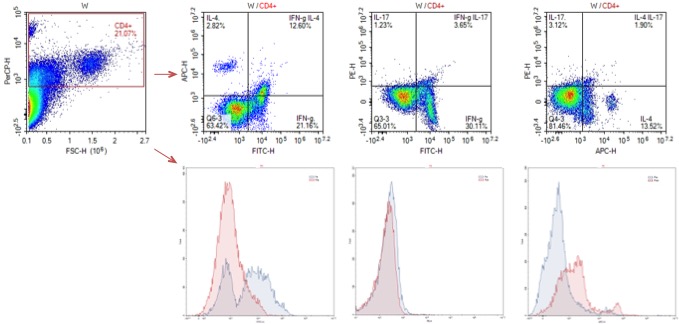


Supplementary Table 1: Data of total amount (cc/μL) of lymphocytes subpopulations of pre- and post-exposure samples. Data have been obtained with the formula:

$$cc/\mu L=\frac{\left( \frac{\mathrm{count}}{volume analyzed} \cdot final volume of cell suspension \right)}{initial blood volume}$$

Being the volume analyzed by the flow cytometer 100 μL, the final volume of cell suspension 2 mL for CD3^+^, CD4^+^, and CD8^+^ analysis and 500 μL for CD4^+^ IFN-γ^+^ IL-4^+^ IL-17^+^, and 200 μL the initial blood volume. The statistical analysis was performed through Wilcoxon test for non-parametric distribution and paired t-test for parametric distribution (for CD3^+^CD8^+^ only), with *p ≤ 0.05, **p ≤ 0.01, ***p ≤ 0.001, ****p ≤ 0.0001.

|  | | Pre | | | Post | | |
| --- | --- | --- | --- | --- | --- | --- | --- |
|  | Mean (± SD) | | Median (Min; Max) | Mean (± SD) | | Median (Min; Max) |  |
| CD3^+ *^ | 599 (± 740.77) | | 473.75 (6.70; 4095) | 430.90 (± 384.82) | | 362.10 (191.30; 2244) |  |
| CD3^+^CD4^+ **^ | 254.78 (±115.12) | | 218 (103; 626.90) | 198.55 (± 82.79) | | 182.95 (62.50; 394.50) |  |
| CD3^+^CD8^+ ***^ | 165.32 (± 76.62) | | 152.50 (39.80; 325.80) | 116.20 (± 49.81) | | 110.65 (40.10; 250) |  |
| CD3^+^CD4^+^CD8^+^ | 4.02 (±5.88) | | 1.75 (0.50; 27.40) | 5.53 (± 4.74) | | 3.40 (0.90; 17.40) |  |
| CD4^+^IFN-γ^+^ | 54.94 (± 57.97) | | 55.85 (1.05; 176.80) | 58.12 (± 37.87) | | 43.60 (17.80; 161) |  |
| CD4^+^IL-4^+ ****^ | 9.88 (± 11.89) | | 7.54 (0.45; 48.83) | 31.67 (± 16.50) | | 27.50 (11.23; 77.38) |  |
| CD4^+^IL-17^+ *^ | 5.61 (± 4.47) | | 3.18 (1.55; 16.35) | 8.51 (± 4.59) | | 8.35 (0.75; 18.30) |  |
| CD4^+^IFN-γ^+^IL-4^+ ****^ | 5.27 (±10.19) | | 1.35 (0.33; 42.75) | 23.52 (± 16.60) | | 19.25 (4.33; 69) |  |
| CD4^+^IFN-γ^+^IL-17^+^ | 2.98 (±3.08) | | 1.49 (0.35; 11.33) | 2.99 (± 1.82) | | 2.93 (0.25; 7.38) |  |
| CD4^+^IL-4^+^IL-17^+ ***^ | 0.58 (±0.73) | | 0.36 (0.05; 3.43) | 1.68 (±1.26) | | 1.54 (0.15; 4.53) |  |
| CD4^+^IFN-γ^+^/  CD4^+^IL 4^+ ****^ | 5.21 (±3.41) | | 5.01 (0.56; 12.98) | 1.86 (± 0.74) | | 1.60 (0.84; 4.32) |  |

Supplementary Table 2: Statistically significantly modulated miRNAs following glyphosate exposure, obtained from QuantStudio 12K Flex OpenArray® system performed on 3 pre- and 3 post-exposure blood samples. The target miRNA, the fold change (2^-ΔΔCt^) and the P value are shown.

| miRNA | 2^-ΔΔCt^ | P value |
| --- | --- | --- |
| hsa_miR-424 | 2.210 | 0.001 |
| hsa_miR-597 | 0.781 | 0.006 |
| hsa_miR-1291 | 5.598 | 0.007 |
| hsa_miR-1249 | 2.203 | 0.012 |
| hsa_miR-27a | 1.436 | 0.014 |
| hsa_miR-10b | 2.699 | 0.017 |
| hsa_miR-100 | 1.757 | 0.017 |
| hsa_miR-500a | 1.579 | 0.023 |
| hsa_miR-301 | 0.476 | 0.023 |
| hsa_miR-374 | 0.534 | 0.030 |
| hsa_miR-136 | 3.042 | 0.040 |
| hsa_let7f | 0.450 | 0.048 |

Supplementary Table 3. Raw data of the concentrations (pg/ml) of the plasma cytokines in the subjects pre- and post-exposure, obtained from ELISA commercial kits.

| Subject | Pre-exposure | Post-exposure | Subject | Pre-exposure | Post-exposure |
| --- | --- | --- | --- | --- | --- |
| IL-4 |  |  | **IL-5** |  |  |
| #1 | 11.1 | 21.2 | #1 | 36.4 | 91.0 |
| #2 | 15.4 | 22.8 | #2 | 45.2 | 91.0 |
| #3 | 16.2 | 21.2 | #3 | 23.9 | 82.8 |
| #4 | 13.8 | 21.2 | #4 | 26.9 | 75.2 |
| #5 | 16.2 | 28.2 | #5 | 25.6 | 61.4 |
| #6 | 23.8 | 32.6 | #6 | 25.6 | 75.2 |
| #7 | 15.4 | 30.4 | #7 | 20.1 | 75.2 |
| #8 | 11.7 | 26.4 | #8 | 22.2 | 82.8 |
| #9 | 15.4 | 19.6 | #9 | 57.3 | 67.8 |
| #10 | 13.8 | 18.0 | #10 | 51.5 | 75.2 |
| #11 | 15.4 | 19.6 | #11 | 30.1 | 82.8 |
| #12 | 15.4 | 21.2 | #12 | 30.1 | 44.2 |
| #13 | 19.8 | 28.4 | #13 | 45.2 | 49.4 |
| #14 | 19.8 | 24.6 | #14 | 32.8 | 44.2 |
| #15 | 18.8 | 28.4 | #15 | 36.4 | 49.4 |
| #16 | 11.7 | 19.6 | #16 | 30.1 | 55.2 |
| #17 | 16.2 | 22.8 | #17 | 41.1 | 75.2 |
| #18 | 17.0 | 24.6 | #18 | 41.9 | 75.2 |
| #19 | 16.2 | 19.6 | #19 | 31.4 | 91.0 |
| #20 | 16.2 | 22.8 | #20 | 50.6 | 82.8 |
| #21 | 19.8 | 28.4 | #21 | 17.6 | 99.8 |
| #22 | 13.8 | 19.6 | #22 | 23.9 | 61.4 |
| #23 | 17.0 | 22.8 | #23 | 23.3 | 49.4 |
| #24 | 16.2 | 21.2 | #24 | 29.4 | 49.4 |
| #25 | 19.8 | 22.8 | #25 | 55.3 | 39.4 |
| #26 | 17.9 | 19.6 | #26 | 34.2 | 61.4 |
| IL-8 |  |  | **IL-12** |  |  |
| #1 | 8.8 | 4.4 | #1 | 125.5 | 106.2 |
| #2 | 8.1 | 3.0 | #2 | 193.0 | 109.0 |
| #3 | 9.6 | 3.4 | #3 | 79.3 | 38.2 |
| #4 | 8.1 | 2.8 | #4 | 249.8 | 112.0 |
| #5 | 8.5 | 5.2 | #5 | 120.6 | 92.6 |
| #6 | 11.3 | 4.4 | #6 | 58.5 | 35.4 |
| #7 | 6.4 | 7.0 | #7 | 285.6 | 214.4 |
| #8 | 5.0 | 4.2 | #8 | 176.7 | 80.2 |
| #9 | 7.7 | 7.0 | #9 | 22.8 | 20.2 |
| #10 | 6.4 | 4.4 | #10 | 25.9 | 28.2 |
| #11 | 7.7 | 3.0 | #11 | 209.6 | 148.0 |
| #12 | 8.5 | 3.0 | #12 | 243.9 | 140.8 |
| #13 | 8.8 | 3.4 | #13 | 243.9 | 287.8 |
| #14 | 9.6 | 4.2 | #14 | 88.1 | 109.0 |
| #15 | 9.6 | 4.8 | #15 | 166.1 | 167.0 |
| #16 | 6.7 | 7.0 | #16 | 496.3 | 675.6 |
| #17 | 7.4 | 3.0 | #17 | 393.7 | 520.0 |
| #18 | 7.7 | 7.0 | #18 | 374.0 | 800.2 |
| #19 | 7.1 | 3.0 | #19 | 243.9 | 115.0 |
| #20 | 6.7 | 3.0 | #20 | 150.5 | 95.2 |
| #21 | 8.1 | 7.0 | #21 | 711.1 | 100.6 |
| #22 | 7.4 | 6.4 | #22 | 54.6 | 38.2 |
| #23 | 7.7 | 5.6 | #23 | 226.6 | 191.8 |
| #24 | 8.5 | 4.4 | #24 | 70.8 | 52.0 |
| #25 | 8.5 | 5.8 | #25 | 273.5 | 293.6 |
| #26 | 10.0 | 3.6 | #26 | 97.1 | 67.0 |
| IL-17 |  |  | **IL-33** |  |  |
| #1 | 5.6 | 24.3 | #1 | 26.9 | 42.6 |
| #2 | 5.6 | 19.7 | #2 | 17.9 | 29.4 |
| #3 | 5.8 | 7.2 | #3 | 35.2 | 12.0 |
| #4 | 5.8 | 4.8 | #4 | 11.3 | 13.0 |
| #5 | 5.2 | 5.7 | #5 | 14.0 | 19.0 |
| #6 | 11.0 | 20.8 | #6 | 19.3 | 17.8 |
| #7 | 5.6 | 4.9 | #7 | 25.9 | 59.0 |
| #8 | 5.2 | 6.6 | #8 | 9.4 | 20.2 |
| #9 | 6.2 | 4.9 | #9 | 34.1 | 15.8 |
| #10 | 5.8 | 8.3 | #10 | 33.0 | 14.6 |
| #11 | 5.6 | 4.1 | #11 | 25.9 | 12.0 |
| #12 | 6.2 | 4.1 | #12 | 14.6 | 11.2 |
| #13 | 5.6 | 7.2 | #13 | 17.9 | 13.8 |
| #14 | 6.4 | 6.2 | #14 | 7.4 | 13.8 |
| #15 | 4.6 | 3.1 | #15 | 13.4 | 13.8 |
| #16 | 6.2 | 3.7 | #16 | 13.4 | 15.6 |
| #17 | 5.8 | 4.1 | #17 | 40.1 | 16.6 |
| #18 | 5.8 | 4.4 | #18 | 49.7 | 13.0 |
| #19 | 6.2 | 2.8 | #19 | 30.8 | 15.6 |
| #20 | 6.2 | 4.8 | #20 | 27.8 | 13.8 |
| #21 | 6.2 | 5.7 | #21 | 22.4 | 13.8 |
| #22 | 6.4 | 4.8 | #22 | 17.2 | 14.6 |
| #23 | 5.8 | 4.8 | #23 | 23.3 | 12.8 |
| #24 | 6.2 | 5.7 | #24 | 12.3 | 17.8 |
| #25 | 6.6 | 6.6 | #25 | 59.2 | 14.6 |
| #26 | 6.4 | 3.7 | #26 | 59.2 | 12.0 |
| IFN-γ |  |  |  |  |  |
| #1 | 17.2 | 40.6 |  |  |  |
| #2 | 13.6 | 44.8 |  |  |  |
| #3 | 12.2 | 29.6 |  |  |  |
| #4 | 14.1 | 36.6 |  |  |  |
| #5 | 72.9 | 279.2 |  |  |  |
| #6 | 25.4 | 36.6 |  |  |  |
| #7 | 25.4 | 67.2 |  |  |  |
| #8 | 12.2 | 51.6 |  |  |  |
| #9 | 12.7 | 33.2 |  |  |  |
| #10 | 11.4 | 56.4 |  |  |  |
| #11 | 12.2 | 23.6 |  |  |  |
| #12 | 16.1 | 26.6 |  |  |  |
| #13 | 43.2 | 33.2 |  |  |  |
| #14 | 17.8 | 33.2 |  |  |  |
| #15 | 16.1 | 40.6 |  |  |  |
| #16 | 118.7 | 554.4 |  |  |  |
| #17 | 15.1 | 35.0 |  |  |  |
| #18 | 26.8 | 99.6 |  |  |  |
| #19 | 15.6 | 25.0 |  |  |  |
| #20 | 17.8 | 31.4 |  |  |  |
| #21 | 19.5 | 26.6 |  |  |  |
| #22 | 13.1 | 17.4 |  |  |  |
| #23 | 16.1 | 35.0 |  |  |  |
| #24 | 12.7 | 22.2 |  |  |  |
| #25 | 18.3 | 25.0 |  |  |  |
| #26 | 18.9 | 26.6 |  |  |  |
